# High-resolution, real-time exercise stress cine

**DOI:** 10.1186/1532-429X-16-S1-W14

**Published:** 2014-01-16

**Authors:** Rizwan Ahmad, Samuel T Ting, Jason Craft, Shivraman Giri, Ning Jin, Subha V Raman, Orlando P Simonetti

**Affiliations:** 1 Ohio State University, Columbus, Ohio, USA; 2 Siemens Healthcare, Columbus, Ohio, USA

## Background

Despite the technological advances made in the field of MRI, further improvements in both data acquisition and processing are required to expand the reliability and diagnostic accuracy of challenging CMR applications such as real-time stress imaging. Due to exaggerated breathing motion and high heart rates, real-time stress images are limited in terms of resolution and often exhibit significant artifacts. In this work, we combine a recently proposed method for variable density incoherent spatiotemporal sampling, called VISTA [1], and SPIRiT-based reconstruction [2] with 3D spatiotemporal regularization to reconstruct real-time stress cine images.

## Methods

Rest and stress (Bruce protocol) cine images were acquired from three healthy volunteers using a 1.5 T (Avanto, Siemens) scanner with 32-channel body coil array. The acquisition was carried out under free-breathing conditions in both short and long axis orientations. The data were collected using four acceleration rates (R = 4, 6, 8, and 10) and two sampling patterns: traditional uniform interleaved sampling (UIS), and VISTA. Other imaging parameters included: 48 frames, 192 × 128 matrix size, 360 × 292 mm2 FOV, 8 mm slice thickness, and maximum temporal resolution of 32 ms (at rate 10). For VISTA sampling, a nonlinear conjugate gradient method was used to perform SPIRiT-based reconstruction, with the SPIRiT kernels estimated from the fully-sampled, time-averaged data. To exploit the spatiotemporal structure in the image, l1-regularization in the 3D discrete wavelet domain was employed. All data were reconstructed offline in MATLAB (version 2013b) using an Intel Core i5 workstation with 24 GB memory. Signal to noise ratio (SNR)--defined as the inverse of the standard deviation of pixel intensities over peripheral regions devoid of NMR signal--was also measured.

## Results

**Figure 1:**
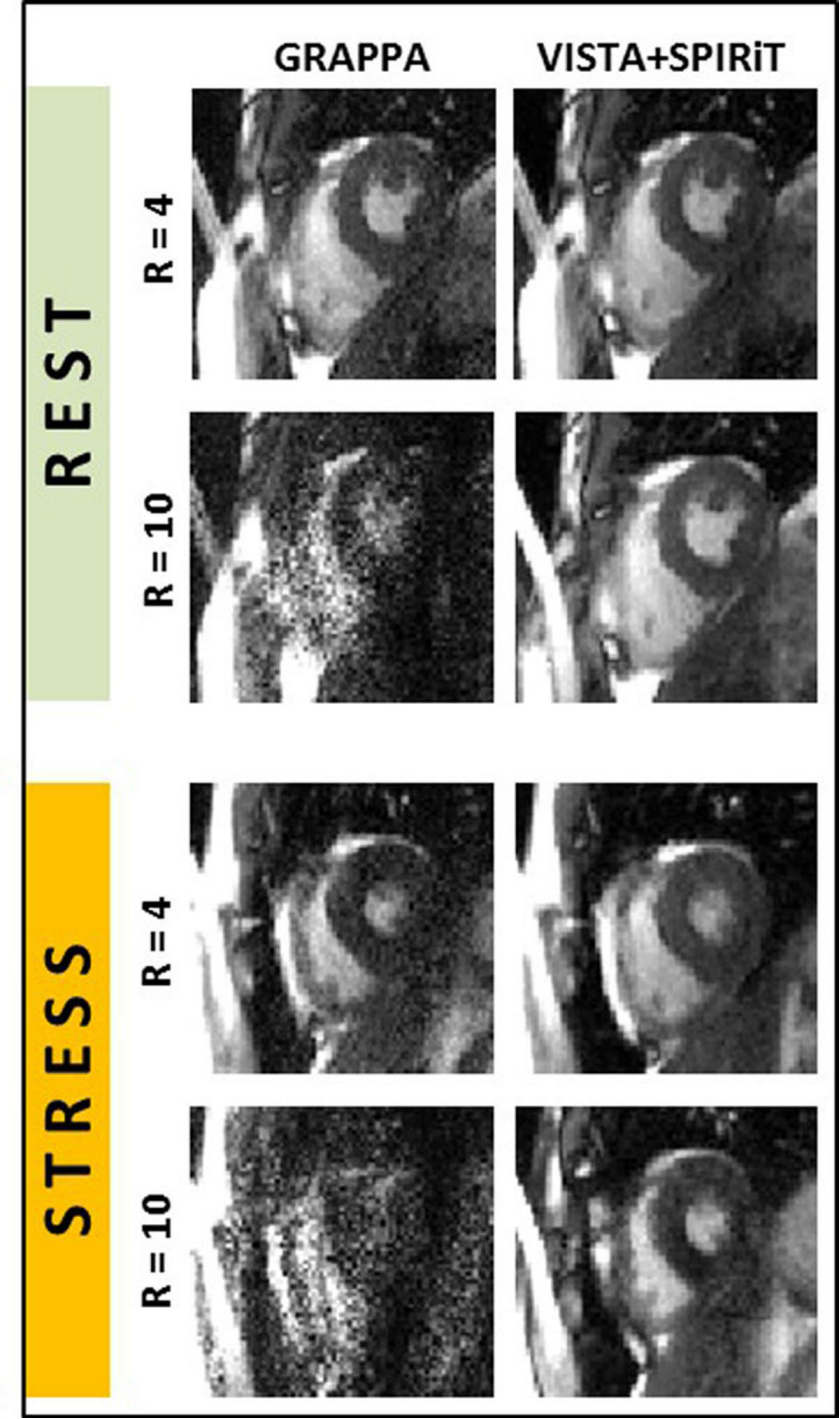
**Real-time, free-breathing cine images under rest and stress conditions**. The proposed VISTA+SPIRiT is compared against traditional GRAPPA reconstruction; the results at two different acceleration rates are displayed.

Figure 1 shows example images at acceleration rates of 4 and 10 using the traditional UIS sampling and GRAPPA reconstruction (UIS+GRAPPA) and the incoherent VISTA sampling and l1-regularized SPIRiT (VISTA+SPIRiT). Compared to the traditional UIS+GRAPPA approach, VISTA+SPIRiT results showed 3 to 5-fold improvement in SNR. Visually, VISTA+SPIRiT also shows minimum artifacts compared to UIS+GRAPPA, especially at high acceleration rates. For VISTA+SPIRiT, the reconstruction time for one dataset was under 30 minutes.

## Conclusions

Combination of VISTA and SPIRiT with spatiotemporal l1-regularization allows high quality images at sub 35-ms temporal resolution and sub 2.5-mm spatial resolution under conditions of high heart rates and exaggerated breathing motion following exercise stress.

## Funding

The work was funded by R01 HL102450 from the NIH.
